# Incidence and Predictive Factors for Surgical Interventions Following Simple Congenital Heart Disease Interventional Transcatheter/Interventional Procedure

**DOI:** 10.3390/jcdd13050217

**Published:** 2026-05-18

**Authors:** Yao Deng, Minzhang Zhao, Xiaoyu Zhang, Chunjie Mu, Runwei Ma

**Affiliations:** Department of Cardiac Surgery, Fuwai Yunnan Hospital, Chinese Academy of Medical Sciences/Affiliated Cardiovascular Hospital of Kunming Medical University, Kunming 650032, China

**Keywords:** congenital heart disease, interventional occlusion procedures, predictive factors

## Abstract

Background: Interventional occlusion procedures for congenital heart disease (CHD) carry the risk of complications requiring reintervention, yet predictive factors remain unclear. Methods: This retrospective case–control study included patients (*n* = 4190) with simple CHD who underwent transcatheter/interventional procedure (2017–2022). Perioperative and postoperative complications were monitored at 1, 3, and 6 months after occlusion. Among them, 44 patients required reintervention for complications. Statistical analysis was performed on clinical data, ultrasound findings from various locations, and laboratory examination results. Results: For atrial septal defects (ASD), independent predictors were defect size and age grading, while those for ventricular septal defects (VSD) were occluder device size, aortic annulus inner diameter, body surface area class, and whether the defect was isolated. The areas under the curve (AUC) of the receiver operating characteristic (ROC) curve for patients who experienced severe complications requiring surgical repair according to ASD were 0.723, whereas for VSD, the AUCs for occluder device size and aortic valve annulus diameter among patients who experienced severe complications requiring surgical repair were 0.649 and 0.539, respectively. Conclusions: This study provides an inaugural comprehensive analysis of occurrence rates and predictive factors for severe post-interventional occlusion procedure complications requiring reintervention. These findings offer new insights as a reference for the treatment of CHD.

## 1. Introduction

Congenital heart disease (CHD) can be classified into simple and complex types based on its anatomical and physiological characteristics and the level of intervention required for its treatment. Simple CHD typically refers to relatively minor or isolated structural heart abnormalities with mild functional impairment, often amenable to interventions such as occlusion procedures. Examples include atrial septal defects (ASD), patent foramen ovale (PFO), ventricular septal defects (VSD), and patent ductus arteriosus (PDA) [1]. As interventional occlusion techniques continue to advance and occlusion materials undergo development, they have progressively become the preferred treatment modalities for simple CHD. However, despite these advancements, complications of varying degrees can occur due to individual patient variations, improper selection of surgical indications, or inadequate technical proficiency.

Moreover, severe complications may require further intervention. Nevertheless, at present, instances of repeat intervention following interventional occlusion procedures are predominantly found in individual case descriptions, and systematic research on the predictive factors for these interventions remains limited. Therefore, this study aimed to investigate the incidence, treatment outcome, and predictive factors associated with reintervention after interventional occlusion procedures for simple CHD, focusing on strategies to prevent and manage severe complications, thereby reducing the need for subsequent intervention.

## 2. Materials and Methods

### 2.1. Patient Population

We conducted a retrospective analysis of 4190 patients who underwent transcatheter/interventional procedures at Fuwai Yunnan Cardiovascular Hospital from September 2017 to September 2022.

**Figure 1:**
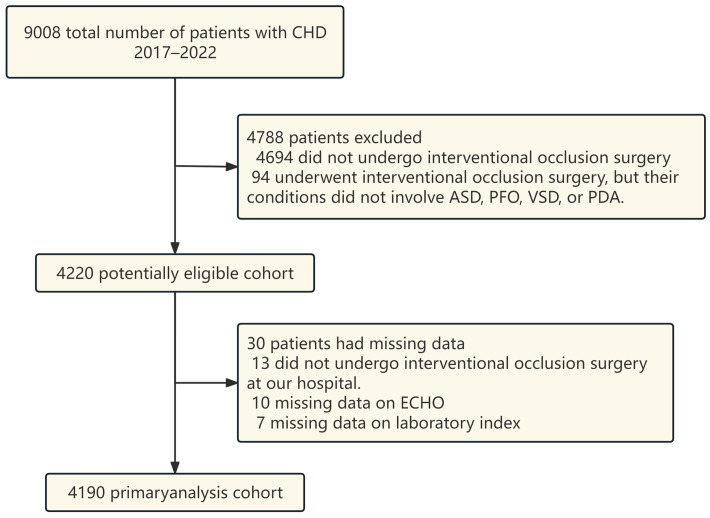
Flow diagram of selected patients, CHD; Congenital heart disease; ASD, Atrial septal defect; PFO, Patent foramen ovale; VSD, Ventricular septum defect; PDA, Patent ductus arteriosus; ECHO, Echocardiogram.

The data selection process was strictly standardized to ensure the reliability and representativeness of the study cohort, with eligible patients retrospectively selected from the electronic medical record system of our hospital during the aforementioned study period. A total of 9008 patients with CHD were diagnosed at our hospital. However, 4818 patients were excluded from the statistical analysis because they did not undergo a transcatheter/interventional procedure, did not receive it at our hospital, or had incomplete clinical data. Detailed information regarding patient screening and exclusion is shown in Figure 1. Ultimately, 4190 patients met the inclusion criteria and were included in the analysis. All enrolled patients were further classified into three groups, namely the CC− group, CC+ group, and CC++ group, based on the occurrence of complications during the perioperative period or follow-up, as well as the need for further intervention due to severe complications. The specific definitions of the three groups were as follows: CC− group: Patients without any complications during the perioperative period and follow-up; CC+ group: Patients who developed complications but did not receive any clinical intervention; CC++ group: Patients who developed severe complications that required further clinical intervention. To ensure the rigor of patient screening, the inclusion and exclusion criteria were clearly defined and strictly implemented, as follows:

Inclusion Criteria: (1) A confirmed diagnosis of CHD was made by preoperative transthoracic echocardiography (TTE), which included a detailed assessment of cardiac structure, hemodynamic parameters, and defect characteristics. (2) Patients underwent interventional occlusion procedures to correct hemodynamic abnormalities caused by CHD, with the aim of improving cardiac function and clinical outcomes.

Exclusion Criteria: (1) Patients who did not undergo interventional occlusion procedures in our hospital, as off-site surgical records could not be fully verified and followed up. (2) Incomplete clinical data, including missing preoperative TTE reports, laboratory test results (e.g., blood routine, coagulation function, liver and kidney function), intraoperative records, or postoperative follow-up information. (3) CHD types not suitable for interventional occlusion, including those not involving ASD, VSD, PFO, or PDA. (4) Complex CHD accompanied by severe pulmonary hypertension or significant cardiac structural abnormalities that could affect the safety and effectiveness of interventional occlusion. (5) Concurrent presence of other diseases that might interfere with surgical outcomes, such as active infections, coagulation disorders, severe liver or kidney dysfunction, or malignant tumors.

### 2.2. Baseline Data Collection

Consistent with the retrospective design, all data were collected via retrospective review. This single-center study ensured uniform interventional procedures by the same specialist team, eliminating operational variations and enhancing result reliability. Differentiated occluder selection strategies were adopted for different CHD types. Clinical data were systematically collected by disease type, including sociodemographic data (gender, age, BSA, SBP, DBP, etc.), electrocardiograms, laboratory tests, ultrasound/TTE results, and treatment plans.

1. Clinical Data Collection: Eligible patients’ clinical data, including demographic information, preoperative diagnostic results, intraoperative records, and postoperative short-term outcomes (e.g., discharge criteria and hospital stay), were retrospectively extracted from the hospital’s electronic medical record system. Patients were discharged once their condition stabilized (absence of arrhythmias, hemodynamic stability, and correct occluder positioning), with an average hospital stay of 6 (5, 8) days.
2. Follow-Up Data Collection: All patients were scheduled for outpatient follow-up at 1, 3, and 6 months postoperatively, and follow-up data (including clinical symptoms, TTE findings, laboratory results, and adverse events such as severe complications requiring reintervention) were retrospectively collected from electronic medical records, outpatient logs and follow-up records. Severe complications requiring reintervention (according to interventional occlusion or surgical repair) included device embolization or migration, pericardial tamponade, high degree of atrioventricular block (III-degree atrioventricular conduction block or complete left bundle branch block), mitral or tricuspid valve perforation, and severe residual shunting.

Given the variability in disease types, the choice of the occlusion device varies, and its size is a significant predictive factor for severe complications. We reviewed clinical data of patients with complications or reintervention needs by disease type and conducted comparative analyses with complication-free patients to identify predictive factors. To ensure data reliability (per retrospective study requirements), all collected data (clinical, cost, follow-up) were independently verified and cross-checked by two assessors (Y.D. and Z.X.Y.), with inconsistencies resolved through joint consultation to minimize errors and ensure research rigor.

### 2.3. Definition of Endpoints

Endpoints were defined to evaluate interventional safety, effectiveness, and reintervention predictive factors for ASD, VSD, PFO, and PDA patients:

Primary Endpoint: Incidence of reintervention (additional interventional procedure/surgical repair) within 6 months due to unsatisfactory occlusion, severe complications, or hemodynamic abnormality recurrence. Secondary Endpoints: (1) Reintervention success rate. (2) Incidence of severe complications (device embolization, valve injury, etc.). (3) Reintervention predictive factors (age, CHD type, defect size, etc.).

Endpoint events were determined by three senior cardiologists based on medical records and follow-up data, with uncertainties resolved via collective discussion.

### 2.4. Follow-Up Data and Outcome Assessment

Consistent with the retrospective design, all data were collected via retrospective review. This single-center study ensured uniform interventional procedures by the same specialist team, eliminating operational variations and enhancing result reliability.

Differentiated occluder selection strategies were adopted for different CHD types. Clinical data were systematically collected by disease type, including sociodemographic data (gender, age, BSA, SBP, DBP, etc.), electrocardiograms, laboratory tests, ultrasound/TTE results, and treatment plans.

1. Clinical Data: Retrospectively extracted from electronic medical records, including sociodemographic data, preoperative/postoperative results, intraoperative records, and discharge details (stable condition, average hospital stay 6 (5, 8) days).
2. Follow-Up Data: Collected retrospectively from medical records/outpatient logs for 1, 3, and 6-month follow-ups, including symptoms, TTE findings, and adverse events (severe complications requiring reintervention: device embolization, pericardial tamponade, etc.).

Clinical data of patients with complications/reintervention needs were reviewed by disease type and compared with those of complication-free patients. All data (clinical, cost, follow-up) were independently verified by two assessors (Y.D. and Z.X.Y.), with inconsistencies resolved via consultation.

### 2.5. Quantitative Assessment of Defect Size and Device Size

For ASD, PFO, VSD and PDA, distinct criteria guided the selection of occlusion devices. In cases involving multiple defects, each defect was individually measured, and the cumulative value was used to determine a suitable device size. The selection of occlusion device size is summarized in Table 1 [2,3,4]. Manufacturers of occlusion devices primarily included the HeartR™ Septal Occluder (Beijing Huamedicine Shengjie Technology Co., Ltd., Beijing, China), Shanghai Shape Memory Alloy Septal Occluder (Shanghai Shape Memory Alloy Materials Co., Ltd., Shanghai, China), MemoSorb^®^ Biodegradable Occluder (Lepu Medical Technology (Beijing) Co., Ltd., Beijing, China), and Amplatzer™ Duct Occluder II (AGA Medical Corporation, Plymouth, MN, USA).

### 2.6. Assessment of Complication by Ultrasound

Patients underwent ultrasound assessment, including echocardiography and puncture site ultrasound, from various perspectives and multiple angles to quantify the occurrence of complications. Pericardial effusion was graded as mild (<10 mm), moderate (10–20 mm), or severe (>20 mm) [5]. Residual shunts were classified as micro shunts (<1 mm) or small shunts (1 to 2 mm), with corresponding indications for intervention. Valve damage was classified as micro, small to moderate, or regurgitating [6]. In addition, ultrasound could be used to determine whether the occluder device had experienced embolization or migration.

### 2.7. Assessment of Complications by Electrocardiogram

Patients were monitored using electrocardiograms or ambulatory electrocardiograms preoperatively and postoperatively, with management tailored to address any arrhythmias that arose.

### 2.8. Assessment of Complication by Laboratory Testing

Laboratory test indicators can provide important clues related to infection and hemolysis, but they typically need to be interpreted in conjunction with clinical presentation and other diagnostic results for a definitive diagnosis. Relevant indicators include markers of infection (white blood cell count > 9.5 × 10^9^/L, C-reactive protein > 8 mg/L, neutrophil percentage > 75%) and markers of hemolysis (erythrocyte sedimentation rate: men > 15 mm/h, women > 20 mm/h; hemoglobin < 117 g/L; indirect bilirubin > 15 μmol/L; blood urea nitrogen > 7.1 mmol/L). Detailed results of laboratory parameter analyses are available in Supplementary Tables S1 and S2 and Supplementary Figure S1.

### 2.9. Cost Analysis

Cost data were collected retrospectively from the hospital’s financial management system and medical record system, covering all direct medical costs associated with interventional occlusion procedures and perioperative management. Specific cost components included: (1) Preoperative examination costs, including TTE, electrocardiogram, laboratory tests, and other auxiliary examinations required for preoperative evaluation. (2) Intraoperative costs, such as occluder devices, interventional catheters and instruments, anesthetics, and operating room fees. (3) Postoperative costs, including ward stay fees, medications (antibiotics, antiplatelet drugs, etc.), postoperative re-examinations (TTE, laboratory tests), and nursing care fees.

Cost analysis was conducted from the perspective of the hospital, and all costs were recorded in the local currency, with uniform adjustment for inflation to ensure the comparability of cost data across different time points in the study.

### 2.10. Statistical Analysis

In our dataset, continuous variables following a normal distribution were presented as “mean ± standard deviation“ and compared between groups using an independent sample *t*-test. For variables not following a normal distribution, descriptions were provided as “medians (interquartile ranges), and comparisons between groups were conducted using non-parametric tests. Categorical variables were represented as percentages, and group comparisons were made using the chi-square test. At the same time, for the variables that showed statistical differences, we further used univariate or ordinal logistic regression to evaluate the risk factors associated with complications. The odds ratio (OR) and 95% confidence intervals (CI) were determined. For single-factor indicators, receiver operating characteristic (ROC) curves were constructed, and the area under the curve (AUC) and cutoff values were determined.

Due to the abundance of laboratory data for patients, we employed orthogonal projections to latent structures-discriminant analysis (OPLS-DA) in SIMCA 14.1 (Umetrics, Kinnelon, NJ, USA) to compare preoperative and postoperative data, as well as to screen variables. Subsequently, we integrated the identified differential data with the variables mentioned above for subsequent multivariable or ordered logistic regression analyses.

GraphPad Prism 8 (GraphPad Software, San Diego, CA, USA) was used for graphical analysis, while statistical analysis was conducted using SPSS software, Version 26.0 (IBM Corp., Armonk, NY, USA). The significance level of *p* < 0.05 was considered statistically significant.

## 3. Results

### 3.1. Study Population

In the final cohort of 4190 patients, the average follow-up period was 10.25 months. Among these patients, 44 experienced severe complications necessitating reintervention (CC++ group), 586 developed new complications but did not undergo reintervention (CC+ group), and 3560 remained complication-free during or follow-up (CC− group). In patients who required further interventions, all complications were identified and managed after the initial occlusion procedure, with an average detection time of 0.34 days, as detailed in Table 2.

### 3.2. The Other Results

Thirteen patients who underwent interventional occlusion procedures at external hospitals were excluded. Among them, eight had residual shunts and subsequently underwent repeat interventional occlusion procedures at our hospital, with no abnormalities detected during perioperative or follow-up evaluations. One patient developed a third-degree atrioventricular block, and four had valvular damage, all of which were successfully managed. Due to the importance of ultrasound examination results in decision-making for interventional occlusion procedures and considering that this study revealed a 0.3% complication rate among patients who underwent occlusion procedures at external hospitals, we were unable to access preoperative ultrasound data for these patients. Consequently, these 13 patients who previously underwent interventional occlusion procedures at an external hospital were excluded.

One unique case involved a third-degree atrioventricular block occurring 6 days after the procedure. However, prompt placement of a temporary pacemaker, administration of corticosteroids (prednisolone acetate), polarizing solutions, and aggressive anti-edema therapy were initiated. Following 8 days of treatment, the patient’s heart rhythm normalized, and the temporary pacemaker was removed.

### 3.3. Incidence Outcome of the CC++ Group

The incidence of severe complications requiring intervention during or after the procedure was 1.1% (*n* = 44). Among these, there were 18 instances of ASD, 23 instances of VSD, and 3 instances of PDA. All 44 cases were identified during the perioperative period and promptly managed. Notably, 7 patients underwent re-interventional closure due to device embolization or migration, while 37 patients required intervention. With an incidence rate of 0.3125% for PDA requiring intervention, no comparative listing was deemed necessary. The details of the 35 patients who underwent surgical procedures are shown in Table 3. After undergoing repeat intervention, 44 patients were followed up for an average of 4 months. The follow-up results showed no complications in 42 patients. In addition, one patient still had a small residual shunt after repeat closure intervention, and another patient experienced mild to moderate regurgitation following aortic valve surgical repair.

### 3.4. Echocardiographic Characteristics of the CC++ Group

Multiplane echocardiographic examinations were conducted on 44 patients who underwent repeat intervention due to severe complications following occlusion procedures. Residual shunting was detected in 9 patients, device embolization or migration in 22 patients, and pathological changes in valve function in 14 patients.

### 3.5. Clinical and Procedural Comparison Among the Three Groups

Out of 4190 patients, 1264 patients (30.2%) were younger than 18 years, and 2926 patients (69.8%) were older than 18 years. Moreover, dividing the cohort based on age with a cutoff at 18 years revealed statistically significant differences in the outcomes, as shown in Table 4. Supplementary Table S1 shows a comparison of the clinical characteristics of the three ASD groups. The CC++ group exhibited differences in age grouping, body surface area (BSA) grading, and NYHA grading compared to the other two groups, with no statistically significant difference observed in other clinical features. Supplementary Table S2 shows a comparison of the clinical characteristics of the three VSD groups. This indicates that the CC++ group has a statistically significant difference observed in age grouping and BSA grading. In contrast, no significant differences were noted in other aspects.

During interventional occlusion procedures, it was observed that in ASD cases, the CC++ group exhibited larger defect sizes, right ventricular anterior–posterior diameters, and occluder device size diameters than the CC− group. In VSD cases, patients in the CC++ group tended to have multiple defects, larger defect size, and larger occluder device size.

### 3.6. Independent Predictors of Reintervention

Univariate and ordered logistic regression analyses identified critical predictive factors affecting postoperative severe complications and repeat surgical repair according to risk in patients with ASD and VSD. First, a single-factor logistic analysis was conducted for variables showing significant differences in ASD within the CC++ group, as detailed in Supplementary Table S1. Subsequently, ordered logistic regression analyses were performed on significant factors (*p* < 0.05), including age grouping, defect size, right ventricular anterior–posterior diameter, device size, and thrombocytocrit as presented in Table 5. The same procedure was applied to VSD, and the final factors for ordinal logistic regression analyses included age grouping, BSA grading, defect size, device size, and whether or not the defect was isolated (Table 6). The analysis results revealed that independent predictive factors for patients with ASD requiring reintervention included defect size and age grouping. In contrast, for patients with VSD, the independent predictive factors included device size, aortic annulus inner diameter, BSA grading, Na+ and solitary defect. These findings are important in guiding clinical decisions and improving patient prognosis.

**Figure 2:**
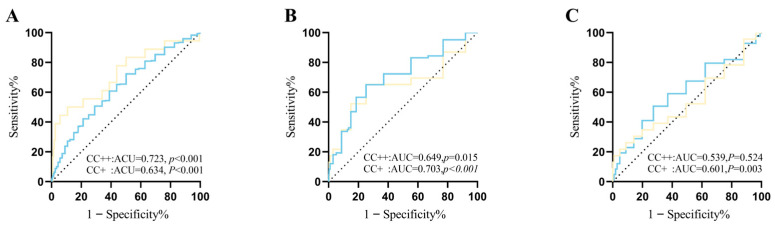
Subject operating characteristic curves showing the ability of defect size to predict postoperative complications after ASD and the ability of occlusion device and aortic annulus inner diameter to predict postoperative complications after VSD. ASD: The Areas under the curve (AUC) for the CC++ group and CC+ group defect sizes (**A**) were 0.723 (*p* < 0.001) and 0.634 (*p* < 0.001), respectively (cutoffs, dark dots). The CC+ group had a defect size >12.40 mm with a sensitivity of 0.654 and a specificity of 0.540; the C++ group had a defect size >21.50 mm with a sensitivity of 0.500 and a specificity of 0.883. VSD: The Areas under the curve (AUC) for device size (**B**) and aortic annulus inner diameter (**C**) were 0.649 (*p* = 0.015), 0.703 (*p* < 0.001), 0.539 (*p* = 0.5241), and 0.601 (*p* = 0.036) for the CC++ group and CC+ group, respectively (cutoffs, dark dots). The CC++ group had a device size of >7.50 mm, sensitivity of 0.522, and specificity of 0.835; aortic annulus inner diameter of >23.5 mm, sensitivity of 0.174, and specificity of 0.985. The CC+ group had a device size of >6.50 mm, sensitivity of 0.566, specificity of 0.795; aortic annulus inner diameter of >16.5 mm, sensitivity of 0.506, specificity of 0.681. The yellow line represents the CC++ group, and the blue line represents the CC+ group. CC+ group: Patients who developed complications but did not receive any clinical intervention; CC++ group: Patients who developed severe complications that required further clinical intervention. ASD: atrial septal defects, VSD: ventricular septal defects, AUC: areas under the curve.

Furthermore, receiver operating characteristic (ROC) curve analysis assessed the diagnostic performance of the risk prediction models for complications in patients with ASD and VSD. For patients with ASD who had not undergone reintervention for complications, the AUC for defect size was 0.634 (95% CI: 0.60–0.66), indicating a moderate level of discriminative ability for the model (Figure 2). The *p*-value was less than 0.001, indicating statistical significance. With a critical threshold set at 12.40 mm, the sensitivity was 0.654, and the specificity was 0.540, implying that when the defect exceeded 12.40 mm, there was a higher probability of increased complication risk. For individuals requiring reintervention after interventional occlusion, the AUC for defect size was 0.723 (95% CI: 0.59–0.86), indicating a better discriminative ability, with a *p*-value also less than 0.001. At a critical threshold of 21.50 mm, the sensitivity decreased to 0.500, whereas the specificity increased to 0.883, suggesting that a larger defect size is associated with a higher risk of surgical repair. In patients with VSD, the AUC for occluder device size was 0.649 (95% CI: 0.51–0.79, *p* = 0.015) in the CC++ group and 0.703 (95% CI: 0.64–0.77, *p* < 0.001) in the CC+ group, both demonstrating significant discriminative ability, with *p*-values less than 0.05, indicating statistical significance. Moreover, the AUC for aortic annulus diameter was 0.539 (95% CI: 0.40–0.68, *p* = 0.524) in the CC++ group and 0.601 (95% CI: 0.53–0.67, *p* = 0.003) in the CC+ group. Notably, in the CC+ group, the result was statistically significant (*p* = 0.003), whereas in the CC++ group, the result was not statistically significant (*p* = 0.524). Further details are shown in Figure 2.

Moreover, the data in Table 5 indicate that among individuals with ASD, adults have a 64% higher risk of developing complications requiring further intervention compared to children, with an OR of 1.64 (*p* = 0.005). Additionally, as per the findings in Table 6, among patients with VSD, individuals with a small BSA face a 3.35 times higher risk of complications than those with a moderate BSA. Regarding defect number, single defects entail a 4.79-fold higher complication risk than multiple defects. To clarify, the total study population included 1.4% (9/627) multiple-defect and 98.6% (618/627) single-defect patients, showing a significant sample size difference between groups.

## 4. Discussion

This single-center retrospective study analyzed the incidence, treatment outcomes, and predictive factors of severe complications requiring reintervention after interventional occlusion for simple congenital heart disease (CHD). A total of 4190 eligible patients who underwent interventional occlusion were enrolled (excluding those with incomplete data) and divided into three groups (CC–, CC+, CC++) based on perioperative complications and reintervention needs. Our results identified key predictive factors for reintervention, providing clinical evidence to support the safe application of interventional occlusion techniques.

### 4.1. Overall Incidence of Reintervention and Baseline Clinical Overview

Among patients with CHD who underwent interventional closure in this study, 1.1% (44 individuals) experienced severe complications and required repeat intervention, while 85% (3560 individuals) did not experience any postoperative complications. Throughout the follow-up period, we additionally confirmed that it was observed that in patients without complications, the closure device maintained a good shape and position, with no adverse effects on heart valve function. Additionally, no fatalities were documented during the entire study duration. This observation corresponds with numerous prior research outcomes, collectively offering substantial evidence supporting the safety and efficacy of interventional occlusion procedures [7,8]. In a single-center study, Varrica A et al. [9] reported that approximately 1.2% of patients required reoperation following a transcatheter/interventional procedure, a proportion similar to our study’s findings. However, unlike our study, they did not delve into potential risk factors through statistical analysis. In contrast, our study further stratified and explored risk factors, respectively, according to different defect types, including ASD and VSD.

### 4.2. Analysis of Reintervention and Risk Factors in ASD

Our results identified key predictive factors for reintervention in ASD patients. The findings of the ROC analysis revealed that an ASD defect size exceeding 12.40 mm was a significant risk factor for patients developing complications without undergoing surgical intervention. Moreover, when the ASD defect size surpasses 21.50 mm, there is a notable increase in the risk of patients experiencing severe complications, necessitating further surgical intervention. Similarly, Cha et al. reported that, as the size of the ASD defect increases, the success rate of transcatheter/interventional procedure proportionally diminishes [10].

Additionally, multiple studies have emphasized that age is another pivotal risk factor for severe complications and the requirement for surgical intervention following interventional ASD occlusion [11,12]. In our study, adults undergoing interventional occlusion had a higher risk of requiring subsequent intervention compared to children. Additionally, complications in adults primarily involved device embolization or migration, with an incidence rate of 77.8% (7/9), whereas children had a lower incidence of 44.4% (4/9). This difference may be attributed to variations in the elasticity and expansibility of surrounding tissues in ASD patients between children and adults. Furthermore, complications following interventional occlusion in children were more diverse, whereas adults tended to experience a more uniform set of complications. Therefore, when considering a transcatheter/interventional procedure for ASD treatment, it is imperative to conduct a comprehensive assessment of both the defect size and age to formulate the most suitable treatment plan.

### 4.3. Analysis of Reintervention and Risk Factors in VSD

Critical factors affecting the need for subsequent surgical intervention after VSD closure were identified in this study, including occluder device size, internal diameter of the aortic valve annulus, the BSA grading, and whether the defect was isolated.

The ROC curve analysis revealed that patients were more susceptible to complications after interventional closure when the occluder size exceeded 6.5 mm. Occluder disk size exceeding 7.5 mm increased the risk of severe complications significantly, requiring further surgical intervention. Moreover, this suggests that as the diameter of the VSD increases, the risk coefficient for subsequent surgical intervention increases. El-Sisi A also proposed that the application of the Amplatzer Duct Occluder II in VSDs should be restricted to smaller defects [13].

Additionally, the diameter of the aortic valve annulus is a pivotal factor in predicting the risk of subsequent surgical intervention. Hence, a precise preoperative measurement of the aortic valve annulus diameter can help predict the likelihood of complications post-transcatheter/interventional procedure. The ROC curve analysis results demonstrated that when the diameter of the aortic valve annulus exceeds 16.5 mm, patients face a corresponding increase in the risk of complications.

BSA is another significant predictive factor. This study revealed that patients with a small BSA had a 3.35 times higher risk of requiring further intervention due to complications than those with a moderate BSA. Although age grouping was not identified as a risk factor for complications requiring further intervention in VSD, the analysis in Table 6 indicates that age differences are statistically significant. Among the 23 patients who experienced severe complications necessitating further intervention, 87% were children (20/23) and 13% were adults (3/23). In adults, the primary complication requiring further intervention was device embolization or migration, occurring in 66.7% (2/3). In children, the probability of device embolization or migration was 50% (10/20). These results are consistent with those observed in ASD.

Furthermore, isolated defects have a 4.79-fold higher complication risk per severity level than multiple defects. Possible reasons are as follows: first, the small sample size of multiple-defect patients (9 cases, 1.4%) may reduce statistical power. A previous study reported a 0.4% (16/4406) prevalence of multiple VSDs [14], consistent with our results, confirming their clinical rarity and potential impact on result stability. Second, clinical management differences contribute: multiple-defect patients, with complex anatomy and possible comorbidities, receive more comprehensive preoperative evaluation, intraoperative monitoring and postoperative care, reducing complications. In contrast, single-defect patients, considered less complex, may receive routine management, leading to inadequate intervention in potential risks and higher complications. Anatomical characteristics also play a role: some single defects may be in key positions (e.g., valve edges), increasing interventional difficulty and complication risk, while multiple defects are often small and non-critical, reducing cardiac impact. Thus, caution is needed when selecting interventional occlusion, especially for single-defect patients requiring careful evaluation and management. This method is less commonly used for multiple-defect patients due to their complex conditions and higher surgical skill requirements.

### 4.4. Common Severe Complications After Interventional Occlusion

The study revealed that major serious complications requiring surgical intervention after closure procedures include residual shunting post-closure, third-degree atrioventricular block, valve damage, device embolization or migration, hemolysis, and infective endocarditis. Embolization or migration of the closure device is the predominant complication, accounting for up to half of all cases.

Based on the procedural details, it is apparent that embolization and migration of the closure device share similar causative factors. These commonly include thin or inadequate margins around the defect, resulting in insufficient support; underestimation of the defect size due to occlusion; selection of a closure device smaller than required; and improper positioning during deployment, exacerbated by factors such as an increased heart rate [15,16]. Typically occurring intraoperatively or within 24 h postoperatively, embolization of the closure device commonly results in its migration into the left or right atrium, and in more severe cases, it may reach the ventricles or pulmonary artery. Once detected by echocardiography, immediate intervention is imperative. If the device can be easily retrieved, reintervention for closure may be attempted; otherwise, urgent reintervention is necessary. As complications arise shortly after dislodgement and the closure device remains unattached, there is little distinction compared to conventional surgical treatment involving thoracotomy. Postoperative follow-up indicated the excellent efficacy of reintervention, with no occurrence of complications.

In this study, after examining pre- and postoperative electrocardiograms, we determined that the overall incidence rate of newly developed arrhythmias was 4.06%, with third-degree atrioventricular blocks accounting for 2.4% of cases. Intraoperative myocardial conduction system edema resulting from device compression is a common factor contributing to the occurrence of arrhythmia. The anatomical relationship between VSDs and the conduction bundle is the primary factor in the occurrence of third-degree atrioventricular block. Mild postoperative arrhythmias typically resolve during follow-up and do not require specific treatment. Symptomatic management may be required in some cases. Numerous studies demonstrate that prompt administration of prednisone therapy can be advantageous in restoring the conduction system for certain severe arrhythmias [17]. Multiple studies have reported that the early onset of new arrhythmias postoperatively is a high-risk factor for the development of severe arrhythmias [18]. However, there are currently no clear guidelines regarding the timely removal of the closure device in cases of early-onset severe arrhythmias postoperatively. In this study, within two days following interventional occlusion, four patients developed arrhythmias. One patient received steroid therapy and temporary pacing, while the other three patients with arrhythmias underwent immediate reintervention. Both treatment methods demonstrated significant efficacy, with no abnormalities observed during the follow-up period. However, the selection of the optimal timing for transcatheter/interventional procedures remains a subject for further investigation, considering individual patient differences.

Multiple studies have shown that hemolysis is related to high-velocity blood flow impacting the metal occluder, resulting in the mechanical rupture of red blood cells [19]. Mild cases may manifest as occult urinary blood, whereas severe cases may present as gross hematuria. In this study, the incidence of hemolysis was 4.55% (2/44), lower than the 8.3% reported in foreign studies [20]. In our study, one patient developed hemoglobinuria accompanied by occluder embolization and mitral valve injury, necessitating immediate open-chest surgical intervention. Another patient exhibited dark-colored urine, urine sediment indicating hematuria, and a decrease in hemoglobin levels postoperatively. Despite the use of sodium bicarbonate to alkalinize the urine, there was no improvement. Given the presence of the occluder, reintervention was performed. Meanwhile, both of these cases showed significant symptom improvement and returned to normal after undergoing surgical intervention to remove the occlusion device. This treatment outcome suggests that timely medical intervention can lead to favorable outcomes for patients even in cases of hemolysis following an interventional occlusion procedure.

Infective endocarditis following interventional closure procedures is less common. Within this study, suspected to be a case: on postoperative day 4, the patient developed an abscess at the left upper arm puncture site with associated rigors. Blood culture on postoperative day 5 confirmed Staphylococcus aureus infection. Ultrasound revealed a tricuspid valve and right coronary artery perforation, necessitating subsequent surgical repair under general anesthesia with hypothermic cardiopulmonary bypass. No intracardiac thrombus formation was observed during this procedure, possibly due to timely postoperative management [21,22]. The pathogenesis may be linked to complications during the endothelialization process of the occluder and concurrent infectious diseases [23]. Hence, thorough postoperative monitoring is essential for patients undergoing a transcatheter/interventional procedure.

### 4.5. Selection of the Closure Device

Advancements in occluder technology for interventional treatment of shunt lesions are pivotal in improving surgical outcomes and reducing postoperative risks for patients. With progress in materials science, occluder materials have evolved from single nickel-titanium alloys to undergo coating treatments for enhanced biocompatibility and reduced thrombotic risks [24]. Subsequently, there has been a gradual transition to utilizing partially or fully biodegradable materials for occluder fabrication [25,26]. These materials can naturally decompose and be absorbed by the body after fulfilling their occlusion task, marking significant development in the field. In terms of design, occluders have progressed from the initial single-disk shape to more intricate double-disk designs [27]. The latter can better conform to the internal structure of the heart, resulting in a more stable occlusion effect. Furthermore, the integration of innovative structures such as waist rings has notably bolstered the stability of occluders, consequently reducing risks during transcatheter/interventional procedures. However, due to cost considerations, these new materials have not yet achieved widespread global adoption.

The HeartR™ Septal Occluder is one of the most frequently employed closure devices in interventional closure. and other occluders demonstrate comparable efficacy [28]. Devices like the HeartR™ Septal Occluder, Beijing Huamedicine Shengjie Technology Co., Occluder, and Shanghai Shape Memory Alloy Septal Occluder are extensively utilized across China due to their cost-effectiveness [28,29]. Similarly, device selection did not influence the occurrence of complications in this study.

### 4.6. Cost-Effectiveness and Superiority

Interventional occlusion is becoming the mainstream approach for the treatment of CHD [30]. Studies have shown that compared with traditional surgical repair, the interventional occlusion procedure not only yields superior treatment outcomes but also, given its shorter hospital stay, offers economic advantages. Our research indicates that the average cost for patients with simple CHD undergoing an interventional occlusion procedure is 24,367.94 yuan, whereas that for surgical repair is 49,724.87 yuan. Therefore, despite the potential risk of requiring further intervention associated with the interventional occlusion procedure, considering the overall medical costs, this procedure remains the preferred treatment option for CHD. Additionally, an analysis by Rigatelli G et al. of the outcomes over the past 20 years of catheter-based or surgical occlusion further demonstrated that patients undergoing interventional occlusion procedures had better outcomes than those undergoing traditional surgical repair, according to [31]. This conclusion further underscores the significance of the interventional occlusion procedure in CHD treatment. Although the interventional occlusion procedure is gaining increasing attention in the treatment of CHD, a thorough assessment remains indispensable for reducing postoperative risks. Traditional surgical repair continues to demonstrate effectiveness in cases of severe complications.

## 5. Limitations

Our study was a retrospective analysis conducted at a single center, with a limited number of patients experiencing severe complications requiring further intervention. Therefore, broader multicenter studies are required to validate our findings. Additionally, although the correlation between defect size, device size, age grouping, BSA grading, aortic valve annulus diameter, and the risk of subsequent surgical repair has been confirmed, a systematic relationship between these factors is yet to be established. Therefore, further studies in this area are warranted.

## 6. Conclusions

The study findings demonstrate that traditional surgical repair is a common and effective approach to managing severe complications arising from interventional occlusion for simple congenital heart disease (CHD), and minimizing subsequent surgical interventions is a key clinical priority to improve patient prognosis. Notably, this study is the first to comprehensively evaluate the effectiveness of repeat interventional procedures and identify relevant predictive indicators, with age being a significant factor—age-related changes in surrounding tissue elasticity affect occluder stability, thereby increasing the risk of complications and reintervention. Comprehensive assessment of age and defect characteristics (e.g., size, rim condition) is therefore crucial for optimal occluder selection, which fulfills the study’s core goal and reflects its innovative value in filling the gap of comprehensive evaluation of repeat intervention effectiveness and predictive factors.

## Figures and Tables

**Table 1:** Selection of occlusion devices for each disease type.

Variable	Concrete Situation
ASD	Children (<18 years old) defect +2~4 mm, adults (>18 years old) defect +4~6 mm.
PFO	Distance from superior vena cava ostium and posterior wall of aortic root for umbrella selection: <12.5 mm, 18 mm; 12.5–17.4 mm, 25 mm; >17.4 mm, 30/35 mm.
VSD	Defect +1~3 mm.
PDA	Inner diameter at narrowest point +3~6 mm (+4~6 mm for infants and children, +2~4 mm for the elderly).

ASD: atrial septal defects, PFO: patent foramen ovale, VSD: ventricular septal defects, PDA: atent ductus arteriosus.

**Table 2:** Complications after interventional closure in patients.

Variable	CC+ Group (*n* = 586)	CC++ Group (*n* = 44)	Total (*n* = 4190)
Residual Shunting (%)	247 (42.15)	9 (20.45)	256 (6.11)
Arrhythmias (%)	167 (28.50)	3 (6.82)	170 (4.06)
Puncture Site (%)	132 (22.53)	-	132 (3.15)
Pericardial Effusion (%)	60 (10.24)	-	60 (1.43)
Valve Damage (%)	25 (4.27)	14 (31.82)	39 (0.93)
Device Embolization or Migration (%)	-	22 (50.00)	22 (0.53)
Hemolysis (%)	-	2 (4.55)	2 (0.05)
Infective endocarditis (%)	-	1 (2.27)	1 (0.02)

CC+ group: Patients who developed complications but did not receive any clinical intervention; CC++ group: Patients who developed severe complications that required further clinical intervention.

**Table 3:** Perioperative conditions for surgical procedures.

Variable	ASD (*n* = 12)	VSD (*n* = 23)	Total (*n* = 35)
Duration of Hospitalization	13 (12.25, 14.75)	14 (12, 17)	14 (12, 17)
ECT (min)	59 (46, 85)	71 (62, 95)	70 (59, 90)
Duration of Aortic Obstruction (min)	31 (19.25, 41.25)	43 (37, 59)	38 (31, 54)
Device Retrieval Procedure (%)	6 (50)	13 (56.5)	19 (54.3)
Defect Repair Procedure (%)	12 (100)	23 (100)	35 (100.0)
Valve Damage			
Aortic valve repairs (%)	-	2 (8.7)	2 (5.7)
Mitral valvuloplasty (%)	1 (8.3)	-	1 (2.9)
Mitral Mechanical Valve Replacement (%)	1 (8.3)	-	1 (2.9)
Tricuspid valvuloplasty (%)	7 (58.3)	3 (13.0)	11
Hemolysis (%)	1 (8.3)	1 (4.3)	2 (5.7)
III-degree atrioventricular block (%)	1 (8.3)	2 (8.7)	3 (8.6)
Infective endocarditis	-	1 (4.3)	1 (2.9)
Preoperative LVEF (%)	65.58 ± 5.55	66.43 ± 4.98	66.00 ± 5.12
Postoperative LVEF (%)	66.83 ± 4.06	63.65 ± 5.45	65.00 ± 5.19
Postoperative ventilator time (h)	5 (4, 8.75)	7 (5, 9)	6 (5, 9)
Postoperative ICU time (h)	53 (19.5, 70.75)	22 (18, 58)	27 (18, 65)

Results are given as the number (percentage), the median (quartiles) or the mean ± SD. ECT: extracorporeal circulation time, LVEF: left ventricular ejection fraction, ASD: atrial septal defects, VSD: ventricular septal defects.

**Table 4:** Age Group Analysis of ASD and VSD.

Variable	<18 Years	≥18 Years	*p*-Value
ASD			<0.001
CC− group, % (*n*/*N*)	85.6% (1233/1441)	78.3% (785/1003)	
CC+ group, % (*n*/*N*)	13.8% (199/1441)	20.8% (209/1003)	
CC++ group, % (*n*/*N*)	0.6% (9/1441)	0.9% (9/1003)	
VSD			<0.001
CC− group, % (*n*/*N*)	85.5% (473/553)	64.9% (48/74)	
CC+ group, % (*n*/*N*)	10.8% (60/553)	31.1% (23/74)	
CC++ group, % (*n*/*N*)	3.6% (20/553)	4.1% (3/74)	

CC– group: Patients without any complications during the perioperative period and follow-up; CC+ group: Patients who developed complications but did not receive any clinical intervention; CC++ group: Patients who developed severe complications that required further clinical intervention. ASD: atrial septal defects, VSD: ventricular septal defects.

**Table 5:** Ordinal logistic regression analysis for the prediction of reoperation for ASD.

Variable	Univariate Analysis		Ordinal Analysis	
	OR (95% CI)	*p*-Value	OR (95% CI)	*p*-Value
Defect Size	1.08 (1.06–1.09)	<0.001	1.07 (1.03–1.11)	<0.001
Device Size	1.06 (1.05–1.08)	<0.001	1.01 (0.97–1.05)	0.564
PCT (%)	0.07 (0.01–0.34)	0.07	0.36 (0.06–2.31)	0.282
Right Ventricular Anterior–Posterior Diameters	1.05 (1.03–1.06)	<0.001	1.00 (0.97–1.02)	0.862
Age Grouping				
<18 years	1.00 (Ref)		1.00 (Ref)	
>18 years	1.64 (1.33–2.03)	<0.001	1.64 (1.16–2.33)	0.005
BSA Grading				
Moderate BSA	1.00 (Ref)		1.00 (Ref)	
Small BSA	1.76 (1.30–2.37)	0.384	1.98 (1.39–2.83)	<0.001
Larger BSA	1.94 (0.93–4.05)	0.005	0.97 (0.68–1.38)	0.864
Maximum BSA	1.27 (0.14–11.37)	0.358	2.11 (0.44–1.80)	0.745
NYHA Grading				
I	1.00 (Ref)		1.00 (Ref)	
II	1.76 (1.30–2.37)	<0.001	1.41 (1.03–1.94)	0.034
II–III	1.94 (0.93–4.05)	0.076	0.94 (0.42–2.12)	0.879
III	1.27 (0.14–11.37)	0.829	0.51 (0.05–4.90)	0.556

PCT: Thrombocytocrit, BSA: body surface area, NYHA: New York Heart Association.

**Table 6:** Ordinal logistic regression analysis for the prediction of reoperation for VSD.

Variable	Univariate Analysis		Ordinal Analysis	
	OR (95% CI)	*p*-Value	OR (95% CI)	*p*-Value
Defect Size	1.24 (1.17–1.31)	<0.001	1.09 (0.99–1.21)	0.091
Device Size	1.24 (1.17–1.32)	<0.001	1.18 (1.06–1.32)	0.003
Internal Diameter of the Aortic Valve Annulus	1.12 (1.06–1.19)	<0.001	1.11 (1.00–1.22)	0.050
Age Grouping				
<18 years	1.00 (Reference)		1.00 (Reference)	
>18 years	2.96 (1.76–4.99)	<0.001	1.30 (0.54–3.10)	0.557
BSA Grading				
Moderate BSA	1.00 (Reference)		1.00 (Reference)	
Small BSA	1.78 (1.02–3.11)	0.042	3.35 (1.70–6.59)	<0.001
Larger BSA	3.40 (1.83–6.31)	<0.001	0.93 (0.38–2.26)	0.865
Maximum BSA	0.00 (0.00–Inf)	-	-	-
Isolated defect				
No	1.00 (Reference)		1.00 (Reference)	
Yes	9.44 (2.80–31.87)	<0.001	4.79 (1.25–18.28)	0.022
Na+	1.93 (1.92–1.94)	<0.001	1.21 (1.08–1.36)	<0.001

BSA: body surface area, OR: odds ratio, CI: confidence intervals.

## Data Availability

The data that support the findings of this study are available from the corresponding author upon reasonable request.

## Supplementary Materials

The following supporting information can be downloaded at: https://www.mdpi.com/article/10.3390/jcdd13050217/s1, Figure S1: Laboratory data analysis in the transcatheter closure pre- and post-procedure by OPLS-DA; Table S1: ASD patients’ characteristics. Table S2: VSD patients’ clinical characteristics.

## Abbreviations

The following abbreviations are used in this manuscript:

CHD	Congenital heart disease
ASD	Atrial septal defects
VSD	Ventricular septal defects
AUC	Areas under the curve
ROC	Receiver operating characteristic
PFO	Patent foramen ovale
PDA	Patent ductus arteriosus
TTE	Transthoracic echocardiography
OR	Odds ratio
CI	Confidence intervals
OPLS-DA	Orthogonal projections to latent structures-discriminant analysis
BSA	Body surface area
ECT	Extracorporeal circulation time
LVEF	Left ventricular ejection fraction
DBP	Diastolic blood pressure
NYHA	New York Heart Association
LYMPH%	Lymphocyte %
PCT	Thrombocytocrit
PLT	Platelet
NEUT#	Neutrophil count,
NEUT%	Neutrophil %
NT-proBNP	N-terminal B-type natriuretic peptide
EO%	Eosinophil %
WBC	White blood cell
