# Supplementary material for: Incidence and Predictive Factors for Surgical Interventions Following Simple Congenital Heart Disease Interventional Transcatheter/Interventional Procedure
Source: J Cardiovasc Dev Dis. 2026 May 18;13(5):217. doi: 10.3390/jcdd13050217 (PMC13207924; doi:10.3390/jcdd13050217)
Supplement: Supplementary file 1 [file jcdd-13-00217-s001.zip › jcdd-4269479-supplementary.pdf]

Supplementary Table S1: ASD patients' characteristics.

Variable	CC- group (n=2018)	CC+ group (n=408)	CC++ group (n=18)	p-value
Clinical characteristics				
Age(years)	13.51(7.14,36.84)b	18.18 (7.69,45.85)	17.76 (7.68,35.31)	<0.001
Sex (%)				0.477
Female	1299(64.4)	275(67.4)	11(61.1)	
Male	719(35.6)	133(32.6)	7(38.9)	
SBP (mmHg)	111(101,121)	112(103,123)	111.5(105.5,122.25)	0.086
DBP (mmHg)	75 (67,82) b	76(69,84)	79(71.5,89.25)	0.003
Body Stature(cm)	150(119,161)	154(123,162)	157(121.5,164.75)	0.124
Body Weight(kg)	42(21.5,56)	48(22.63,58.5)	47(20.75,69.25)	0.046
BSA Grading (%)				0.024
Moderate BSA	581(28.8)	95(23.3)	4(22.2)	
Small BSA	513(25.4)	96(23.5)	4(22.2)	
Larger BSA	866(42.9) b	206(50.5)	8(44.4)	
Maximum BSA	58(2.9) a	11(2.7)	2(11.1)	
NYHA class (%)				<0.001
I	1789(88.7) a	336(82.4)	12(66.7)	
II	199(9.9) a	61(15.0)	6(33.3)	
II ~III	26(1.3)	10(2.5)	-	
III	4(0.2)	1(0.2)	-	
Echocardiography				
Isolated Defect (%)				<0.001
No	192(9.5)	141(34.6)	2(11.1)	
Yes	1826(90.5) b	267(65.4)	16(88.9)	
Left Atrial Anteroposterior Diameter (mm)	12(8,17) ab	16(11,22)	20(12.75,29.25)	<0.001
Left Atrial Anteroposterior Diameter (mm)	25(20,30) b	27(21,33)	28(22.75,31.25)	<0.001
Right Ventricular Anterior-Posterior Diameters (mm)	24(18,30) ab	24(18,30)	30(20.75,36)	<0.001
Procedural parameters				
Device size (mm)	18(14,24) ab	16(11,22)	24(18,38.5)	<0.001
Two devices (%)	18(0.9) b	13(3.2)	-	<0.001
Laboratory features				
LYMPH%	40.60(33.40,48.90)	39.35(32.83,49.80)	38.90(34.68,55.48)	0.820
PCT (%)	0.27(0.23,0.31) b	0.25(0.22,0.30) a	0.31(0.26,0.36)	<0.001
PLT (*109/L)	271(227,326) b	258(212,309)	295(232.5,374)	<0.001
NEUT# (*109/L)	3.00(2.35,3.84)	2.99(2.31,3.82)	3.22(2.46,4.35)	0.646
NEUT%	47.60(39.08,55.43)	49.20(38.83,55.78)	41.90(29.50,54.78)	0.212
NT-proBNP (pg/mL)	63.90(28.73,140.75)	86.32(50.64,284.10)	179.75(62.55,299.60)	0.047

The differences are statistically significant. a: Comparisons were made with the CC++ group, b: Comparisons were made with the CC+ group. This study only investigates the variables that show

differences with the CC++ group, as indicated by the subscript 'a'. Significance values have undergone adjustment for multiple tests through Bonferroni correction, and the Gamma test has been utilized for analyzing age, body surface area grading, and cardiac function grading. SBP: systolic blood pressure, DBP: diastolic blood pressure, BSA: body surface area, NYHA: New York Heart Association, LYMPH%: lymphocyte %, PCT: thrombocytocrit, PLT: platelet, NEUT#: neutrophil count, NEUT%: neutrophil %, NT-proBNP: N-terminal B-type natriuretic peptide.

CC– group: Patients without any complications during the perioperative period and follow-up; CC+ group: Patients who developed complications but did not receive any clinical intervention; CC++ group: Patients who developed severe complications that required further clinical intervention. ASD: atrial septal defects.

Supplementary Table S2: VSD patients' clinical characteristics.

Variable	CC- group (n=521)	CC+ group (n=83)	CC++ group (n=23)	p-value
Clinical characteristics				
Age (year)	7.36(4.20,11.83)b	9.53 (4.24,18.68)	6.09 (3.86,12.80)	0.037
Sex (%)				0.438
Female	275(52.8)	43(51.8)	9(39.1)	
Male	246(47.2)	40(48.2)	14(52.2)	
SBP (mmHg)	109(99,120)	110(102,119) a	98(92,114)	0.031
DBP (mmHg)	71(64,79)	74(67,80)	72(62,85)	0.273
Body Stature(cm)	120(102,145)	131(102,160)	116(91,140)	0.073
Body Weight(kg)	22(16,35)	25(16,50)	19(14,35)	0.081
BSA Grading (%)				<0.001
Moderate BSA	166(31.9) ab	15(18.1)	4(17.4)	
Small BSA	268(51.4)	39(47.0)	15(65.2)	
Larger BSA	81(15.5) b	29(34.9)	4(17.4)	
Maximum BSA	6(1.2)	-	-	
NYHA class (%)				0.165
I	459(88.1)	71(85.5)	17(73.9)	
II	62 (11.9)	12(14.5)	5(21.7)	
II ~III	-	-	1(4.3)	
III	-	-	-	
Echocardiography				
Isolated defect (%)				<0.001
No	3(0.6)	4(4.8)	2(8.7)	
Yes	518(99.4) ab	79(95.2)	21(91.3)	
Defect size (mm)	4(3,6) ab	7(4,10)	8(3,10)	<0.001
Internal Diameter of the Aortic Valve Annulus (mm)	15(13,17) b	17(14,19)	15(13,20)	<0.001
Right Ventricular Anterior-Posterior Diameters (mm)	15(13,18) b	17(14,21)	15(13,20)	<0.001
Procedural parameters				
Device size (mm)	6(5,8) ab	8(6,12)	8(6,12)	<0.001
Laboratory features				
LYMPH%	44.51±12.96	43.24±11.94	44.48±12.86	0.705
Na+ (mmol/L)	138.29±1.71 a	138.87±1.87	141.85±2.41	<0.001
EO%	2.80(1.70,4.60)	2.70(1.60,4.00)	2.30(0.90,3.30)	0.102

Variable	CC- group (n=521)	CC+ group (n=83)	CC++ group (n=23)	p-value
NEUT# (*109/L)	3.01(2.30,4.05)	2.93(2.35,3.67)	2.90(2.16,4.44)	0.799
NEUT%	43.56±13.19	45.19±11.90	44.99±12.77	0.518
WBC (*109/L)	7.19(6.05,8.76) b	6.55(5.77,7.91)	6.74(5.58,8.42)	0.029

The observed differences hold statistical significance. In the context of this comparison, 'a' denotes comparisons with the CC++ group, while 'b' denotes comparisons with the CC+ group. Significance values have undergone adjustment for multiple tests through Bonferroni correction, and the Gamma test has been utilized for analyzing age, body surface area, and cardiac function grading. SBP: systolic blood pressure, DBP: diastolic blood pressure, BSA: body surface area, NYHA: New York Heart Association, LYMPH%: lymphocyte %, EO%: eosinophil %, NEUT#: absolute neutrophil, NEUT%: neutrophil %, WBC: white blood cell.

CC– group: Patients without any complications during the perioperative period and follow-up; CC+ group: Patients who developed complications but did not receive any clinical intervention; CC++ group: Patients who developed severe complications that required further clinical intervention. VSD: ventricular septal defects.

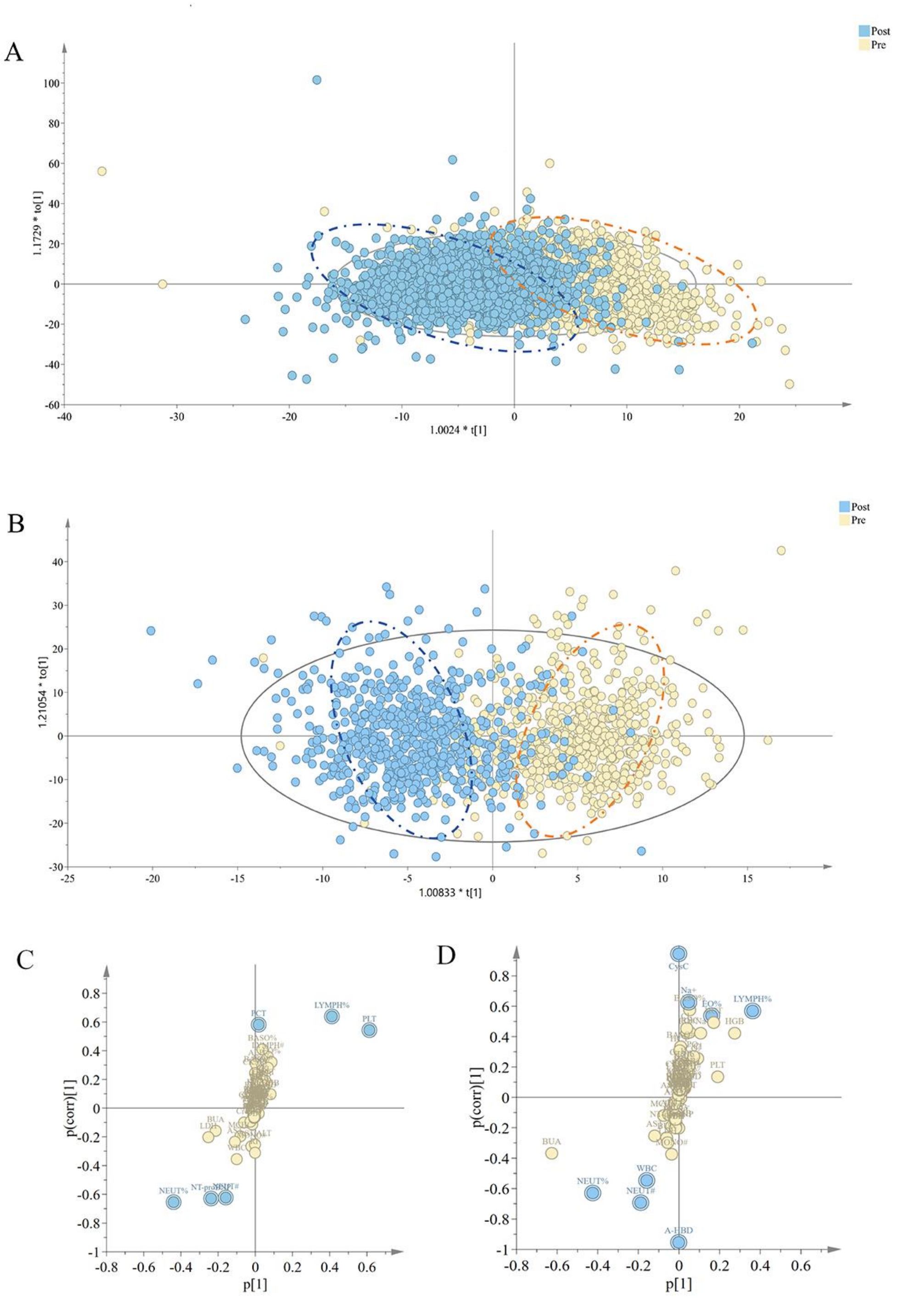

Supplementary Figure S1. Laboratory data analysis in the transcatheter closure pre- and post-procedure by OPLS-DA. A. OPLS-DA score plot of ASD. B. OPLS-DA score plot of VSD. C. S-Plot of ASD. D. S-Plot of VSD. (In C and D, blue is an indicator of difference, and yellow is an indicator of no difference.).

ASD: atrial septal defects, VSD: ventricular septal defects, OPLS-DA: orthogonal projections to latent structures-discriminant analysis.
